# Bearing Capacity and Reinforced Measures of Bolted Joints for Pultruded Composite Square Tubes

**DOI:** 10.3390/ma18132936

**Published:** 2025-06-20

**Authors:** Juan Han, Xinchen Zhang, Zhitian Xie, Hai Fang, Youjun Qi, Wei Song

**Affiliations:** 1 College of Architectural Engineering, Jiangsu Open University, Nanjing 210036, China; hanjuan_060101@163.com; 2 College of Civil Engineering, Nanjing Tech University, Nanjing 211816, China; xc.zhang@njtech.edu.cn (X.Z.); qyj_njut@163.com (Y.Q.); 3 Beijing Key Laboratory of Underground Engineering Construction Prediction & Precaution, Beijing Municipal Engineering Research Institute, Beijing 100037, China; mlfgnr@163.com (Z.X.); songwei170@163.com (W.S.)

**Keywords:** bolted joints, pultruded composite square tubes, reinforcing gasket, bearing capacity

## Abstract

This study investigates the tensile behavior of pultruded composite square tubes with single- and double-bolt joints to evaluate their ultimate load-bearing capacity and failure modes. A series of experiments was carried out to examine the effects of hole size, edge distance, and the presence of a reinforcing gasket on joint performance. The results indicate that incorporating a multiaxial fiber-reinforced resin matrix composite gasket significantly enhances joint strength, achieving up to a 295% increase in bearing capacity. In single-bolt configurations, reducing the edge distance improved the effectiveness of the gasket, leading to higher ultimate strength, as it enhances the lateral confinement of the gasket. For double-bolt joints, a shorter spacing between holes further amplified the reinforcing effect of the gasket under constant hole size and edge distance. A theoretical model was developed to estimate the ultimate load, and the calculated results showed strong agreement with experimental observations. Numerical predictions showed a strong correlation with experimental findings, confirming the model’s reliability and accuracy.

## 1. Introduction

Pultrusion technology is an industrial production process used to manufacture linear composite products with isometric cross-sections. It offers several advantages, including high production efficiency and consistent product quality, when compared to other composite molding processes. Pultruded composite profiles have gained popularity in structural engineering in recent years due to their lightweight nature, high strength, and excellent corrosion resistance [1,2,3,4,5,6,7]. While pultruded glass fiber-reinforced polymer composite profiles can be directly used as structural members, their joints pose challenges due to their brittle behavior [8,9].

**Figure 1:**
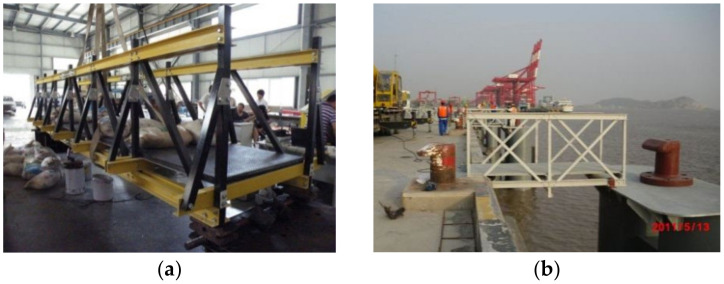
Application of pultruded composite profiles; (**a**) Pultruded composite footbridge; (**b**) Shanghai Yangshan port truss bridge.

In the field of architectural and structural engineering, composite materials have been utilized in various applications. Dicuonzo et al. [10] developed a modular platform using composite materials, while Ahmed Godat et al. [11] applied pultruded composite profile truss structures for transmission support towers. Pultruded composite materials have also been employed in the construction of trussed bridges. For example, a bridge in Greece boasts a span of 12 m and a design load of 30 tons [12], while another one is the Pontresina bridge in the Swiss Alps [13]. In 2012, Tsinghua University designed and constructed the Mao Yisheng public welfare bridge, which features a 40 m-wide pultrusion composite structure. Liu et al. successfully applied a composite pultrusion profile truss bridge in the construction of Shanghai Yangshan port [14] (Figure 1a,b).

The design of joints in pultruded composite profiles is a crucial aspect of composite structures. Bolted, bonded, and hybrid bolted/bonded joint systems are commonly used in composite pultruded structures, with bolted joints being the most prevalent due to their easy disassembly capabilities [15,16]. Bolted connections allow for efficient load transfer and facilitate inspection and repair processes. However, the anisotropy, brittleness, and non-homogeneity of fiber composites contribute to complex failure modes in bolted joints, with damage propagation characteristics and fracture properties differing significantly from those of metal materials. Numerous studies have investigated the mechanical behavior of bolted joints in composite laminates, focusing on failure modes, strength prediction, and the influence of design parameters. Previous work has addressed the effects of laminate properties, fiber orientations, bolt types, and clearance on joint strength and failure mechanisms [17,18,19,20,21,22,23]. However, existing studies mainly focus on single-bolt configurations or simple loading conditions, with limited attention paid to the reinforcing effects of gaskets in multi-bolt joint systems. Sun et al. [24] used experimental and numerical simulation methods to study the effect of bolt pre-tightening forces on the bearing strength of bolt openings. Aktas [25] analyzed the effect of the stacking sequence on the bolt–hole strength. Turvey and Wang [26] studied the bearing capacity of single bolted joints of pultruded glass-reinforced plastics under hygrothermal conditions. The test shows that the bolted-joint strength obviously declines under hygrothermal conditions. Bai et al. [27] proposed a new type of connector made of GFRP pultruded profiles for large-scale space grid structures, which are used to connect chords and slant rods to form composite space trusses. Luo et al. [28] also proposed a bolt-and-sleeve connection method with a tubular section, which applied the composite pultruded profile to the space structure. The research results show that the bearing capacity can be optimized effectively by adjusting the geometry size of the bolted joint. Li et al. [29] proposed a new composite tube connection method called the pre-tightened teeth connection technique to improve the composite tube connection efficiency.

This study presents a comprehensive experimental and numerical investigation of the load-bearing behavior of bolted joints in pultruded composite square tubes. The research systematically evaluates the effects of key geometric parameters—including hole size, edge distance, and bolt spacing—on the tensile performance of single- and double-bolt joints. To enhance joint strength, a multiaxial fiber-reinforced resin matrix gasket is introduced for the first time in such composite connections. These gaskets were selected for their superior multidirectional mechanical properties, which enable more uniform stress distribution and improved load transfer efficiency. Compared with conventional gasket materials, such as metal shims or unidirectional laminates, multiaxial fiber-reinforced gaskets provide enhanced in-plane stiffness, better damage tolerance, and improved bonding compatibility with composite substrates. These characteristics help mitigate local stress concentrations and delay failure onset, particularly under short edge distances or closely spaced bolts. Experimental results confirm that such gaskets significantly improve load capacity and stabilize failure modes. This strengthening strategy is validated through both laboratory tests and finite element simulations using ANSYS/LS-DYNA, showing up to 295% improvement in ultimate bearing capacity. The findings offer a solid theoretical basis for optimizing joint design and promote broader application of pultruded composites in structures such as space trusses.

### 1.1. Test Specimen and Preparation

**Figure 2:**
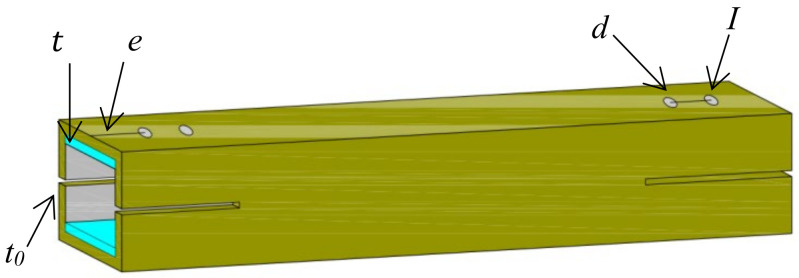
The specimen diagram of the composite pultruded square tube.

In this study, a total of 28 composite pultruded square tubes were manufactured to comprehensively evaluate the effects of bolt diameter, edge distance, and gasket application on joint performance. This number was chosen to ensure adequate coverage of all variable combinations and to support robust comparative analysis while maintaining manageable experimental complexity. Among them, 16 specimens were designed with single-bolt joints and 12 with double-bolt joints. All specimens had identical square cross-sections of 40 × 40 mm, a thickness of 5 mm, and a total length of 300 mm. The details of the single-bolt configurations are listed in Table 1, where the edge distances from the bolt hole to the tube end were set as 1 d to 4 d (with d being the bolt diameter). The edge distances were intentionally selected as multiples of bolt diameter to comply with established design guidelines and to systematically investigate their influence on failure modes—shorter edge distances tend to induce shear-out or splitting failures, while larger ones promote bearing failure. For the double-bolt specimens (see Table 2), the center-to-center spacing between the two bolt holes varied from 2 d to 4 d. A schematic diagram of the specimen layout is provided in Figure 2.

### 1.2. Manufacturing Procedures

The 28 specimens were cut according to the design size from the composite pultruded square tubes. With the same size as the diameter of the bolt, one or two holes were processed at each end of the cut specimens. A solid carbide twist drill bit with a diameter of 10 mm was used for all drilling operations, due to its superior wear resistance and suitability for composite materials.

The gasket was manufactured by three E-glass fiber mats with the upper layer of bi-axial [−45/45], middle layer of bi-axial [0/90], and bottom layer of bi-axial [−45/45] glass fiber (each layer weighing 800 g/m^2^). The outer [−45/45] layers improve shear resistance and confinement around the bolt hole, while the [0/90] core enhances axial and transverse stiffness, forming a balanced and damage-tolerant structure under complex loading. The thickness of each layer was 1 mm, and the total thickness was 3 mm. Three layers of glass fiber fabrics were wrapped in the vacuum bag sealed with the atmospheric pressure and vacuum action, and then the resin was injected into the vacuum bag. After curing the resin for 8 h, the manufacturing process was completed. A two-component epoxy resin was used in this study, which exhibits high mechanical strength, excellent adhesion to glass fibers, and good thermal and chemical resistance, making it suitable for structural composite applications. The total thickness was controlled by the compression effect of the vacuum bagging process to ensure uniform consolidation.

**Figure 3:**
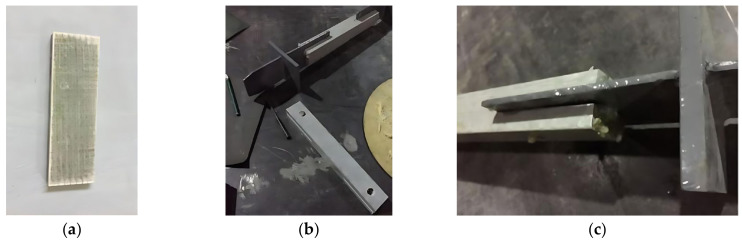
The pasting process of strengthening the gasket; (**a**) Strengthening the gasket; (**b**) Bonding the gasket; (**c**) Clamping the gasket.

Two gaskets were bonded to both ends of each of the 14 specimens using a two-component epoxy structural adhesive. After curing, holes were drilled through the gaskets using an electric drill with the same diameter. The bonding process is shown in Figure 3.

### 1.3. Material Properties

In this study, all mechanical property tests were performed on five replicate specimens to ensure the repeatability and reliability of the results. The tensile and compression tests strictly followed ASTM D3039 [30] and ASTM D695 [31], respectively, with specimen dimensions designed to minimize edge effects and ensure representative material behavior. All coupons were prepared from the same batch of pultruded composite panels to maintain consistency in material quality. Displacement-controlled loading was applied at a rate of 2 mm/min for tension and 1 mm/min for compression, consistent with standard quasi-static conditions.

Table 3 gives an overview of the results. The differences in tensile and compressive moduli between the face sheets and the gaskets may result in stress concentration or strain mismatch near the bolt holes, thereby influencing the local load transfer and failure initiation in the joint.

### 1.4. Experimental Set-Up and Loading Procedure

**Figure 4:**
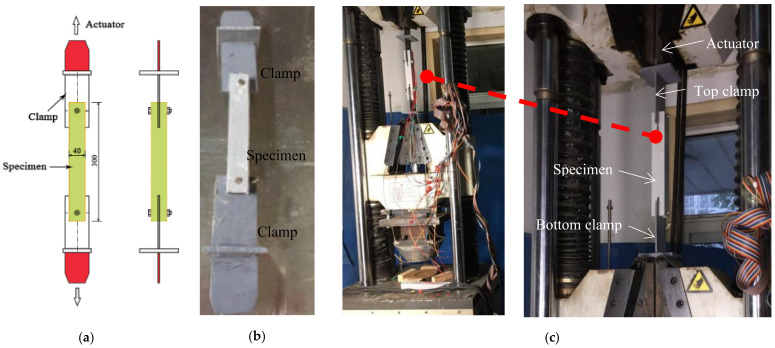
Loading test set-up; (**a**) schematic diagram; (**b**) schematic diagram of the specimen; (**c**) loading test set-up for the specimen.

The experimental set-up can be seen in Figure 4. The tensile–shear tests were conducted using a Zwick/Roell electronic tensile machine with a capacity of 600 kN and data acquisition systems (Ulm, Germany). Each specimen was placed between two rigid plates using a pair of custom-designed metal fixtures to ensure proper alignment. The bottom rigid plate was fixed on the base of the test device, while the top plate was moved vertically upward at a loading rate of 2 mm/min to stretch the specimens. Prior to loading, careful centering was performed to minimize torsional effects or off-axis loading.

## 2. Experimental Results and Discussion

### 2.1. Failure Modes

**Figure 5:**
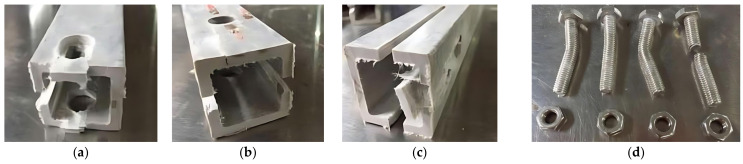
The failure modes of composite pultruded square tubes with single bolt; (**a**) Shear failure; (**b**) Extrusion damage; (**c**) Splitter and extrusion; and (**d**) Bending and cutting failure of bolt.

**Figure 6:**
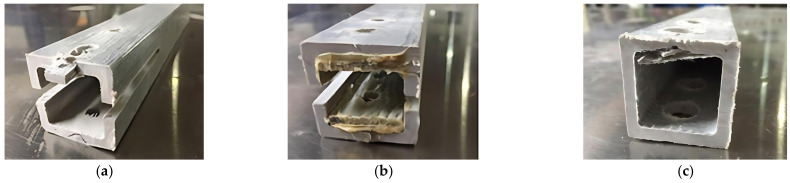
The failure modes of composite pultruded square tubes with double bolts; (**a**) Shear failure at the end of the bolt; (**b**) Extrusion damage between the bolts; and (**c**) Splitter damage at the end of the bolt.

The failure modes of mechanical joints in composite pultruded tubes mainly include tensile failure, shear failure, extrusion failure, splitting failure, bending failure, and shear failure of metal bolts. These modes may occur individually or in combination [15]. The failure pattern is primarily influenced by the joint’s geometric parameters and the fiber orientation of the pultruded tubes. The main failure types observed in all tested specimens are shown in Figure 5 and Figure 6.

**Figure 7:**
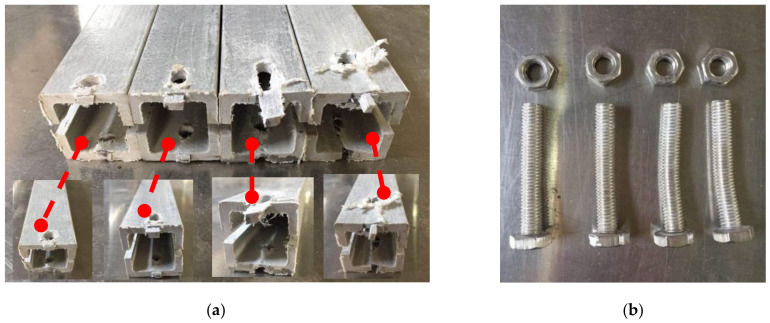
Failure modes of 8 mm diameter specimen for end distance (1 d–4 d); (**a**) without the gasket; (**b**) bolt without the gasket.

**Figure 8:**
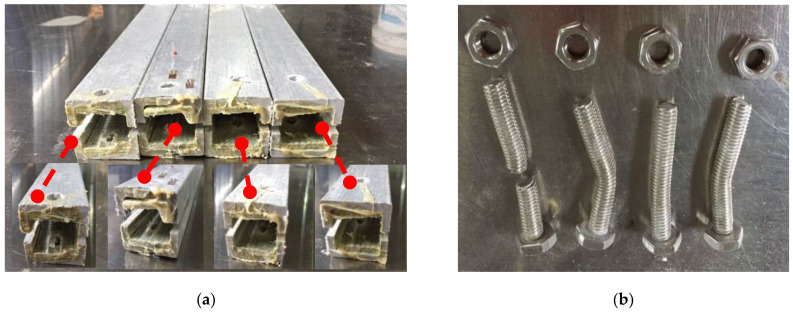
Failure modes of 8 mm diameter specimen with gasket for end distance (1 d–4 d); (**a**) with the gasket; (**b**) bolt with the gasket.

**Figure 9:**
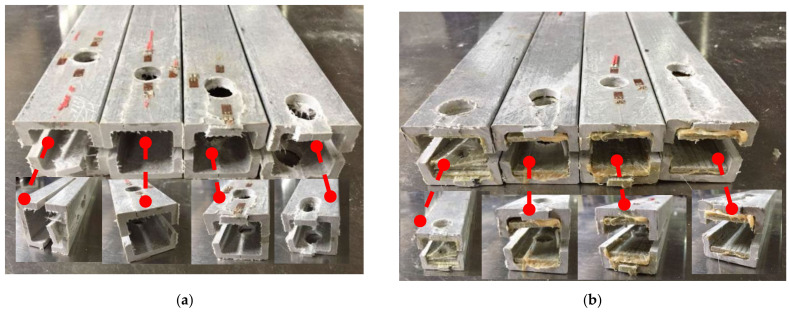
Failure modes for end distance (1 d–4 d); (**a**) without the gasket; (**b**) bolt with the gasket.

**Figure 10:**
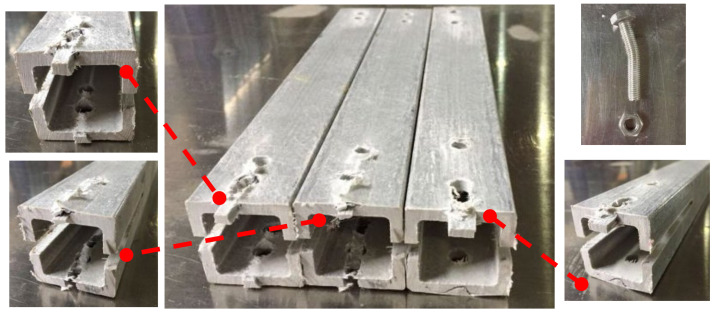
Failure modes of 8 mm diameter double-bolt specimen.

**Figure 11:**
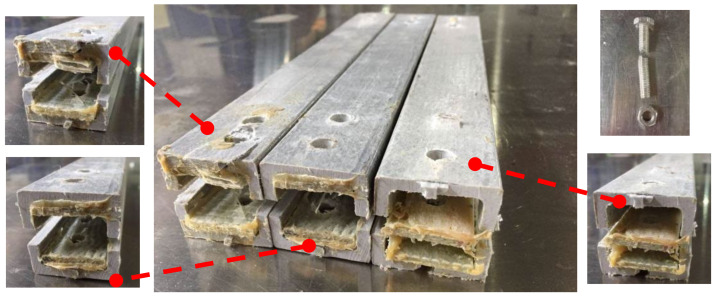
Failure modes of 8 mm diameter double-bolt specimen with gasket.

**Figure 12:**
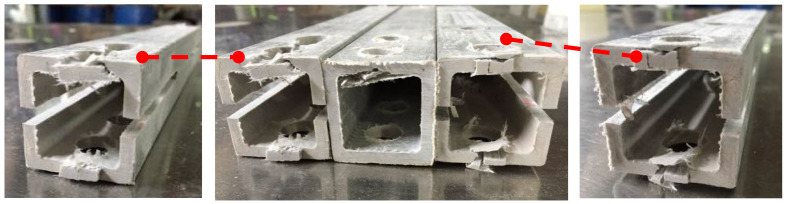
Failure modes of 16 mm diameter double-bolt specimen.

**Figure 13:**
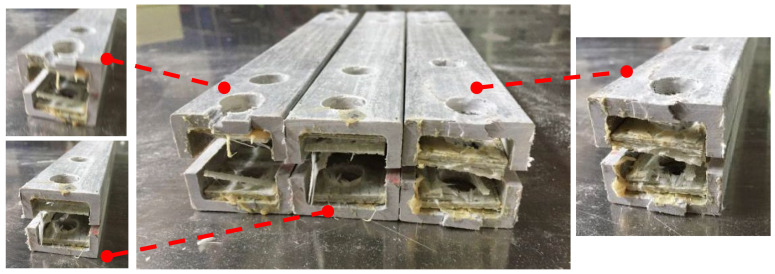
Failure modes of 16 mm diameter double-bolt specimen with gasket.

Shear failure (Figure 5a) was observed in specimens DS-8-1d, DS-8-G-1d, SS-8-2d, and SS-8-G-2d. In these specimens, the material ahead of the bolt hole was extruded along two shear cracks, and the bolt showed slight bending. A combined failure mode of extrusion and shear was found in specimen DS-8-4d (Figure 5b), where a visible crack appeared but no extrusion occurred. Local layer separation resembling a combination of local extrusion and splitting failure was observed at the specimen ends (Figure 6c). Bending failure and shear failure of the metal bolts are shown in Figure 5d. The ultimate loads and failure modes for single-bolt and double-bolt specimens are summarized in Table 4 and Table 5, respectively. Failure modes for all specimens are illustrated in Figure 7, Figure 8, Figure 9, Figure 10, Figure 11, Figure 12 and Figure 13.

These observed failure modes demonstrate that the geometry of the joint and bolt configuration strongly influence the mechanical response of pultruded composite joints. Understanding these failure mechanisms is essential for optimizing joint design and preventing premature failure.

### 2.2. Load and Displacement Relationship

#### 2.2.1. Effect of the Gasket

**Figure 14:**
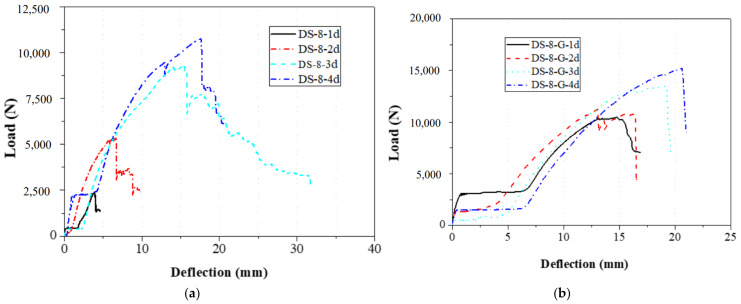
Load–displacement curve of 8 mm diameter specimen with a single bolt; (**a**) without the gasket; (**b**) with the gasket.

**Figure 15:**
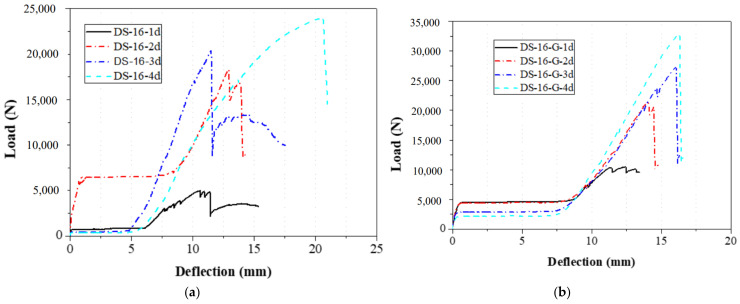
Load–displacement curve of 16 mm diameter specimen with a single bolt; (**a**) without the gasket; (**b**) with the gasket.

The bolt diameter was divided into 8 mm and 16 mm in the tensile and shear tests of single-bolt specimens. During these specimens, the distance from the bolt hole to the end changed from 1 to 4 times the bolt diameter. The load–displacement curves of 8 mm and 16 mm diameter specimens with single bolts are shown in Figure 14 and Figure 15, respectively, and the failure modes are depicted in Figure 7, Figure 8 and Figure 9.

The load–displacement curves of the 8 mm and 16 mm single-bolt specimens showed similar trends: a brief rise, a plateau, a peak, and a linear drop (Figure 14 and Figure 15). The plateau likely resulted from a gap between the bolt and clamp, which was hard to eliminate manually. After reaching the peak, all specimens failed in shear—a brittle mode. Reinforced gaskets significantly improved bearing capacity (up to 294.5%); however, the effect was limited by an eventual separation between the gasket and specimen ends, which was visually observed after failure. At constant bolt diameter, increasing end distance reduced the gasket’s effectiveness. With an 8 mm bolt and 1 d end distance, the load increased by 295% (2.42 kN to 9.56 kN); at 4 d, the increase dropped to 46.95% (10.76 kN to 15.81 kN). For a 16 mm bolt, the improvement was 117.07% (5.51 kN to 11.97 kN) at 1 d, and 24.38% (23.76 kN to 29.56 kN) at 4 d.

**Figure 16:**
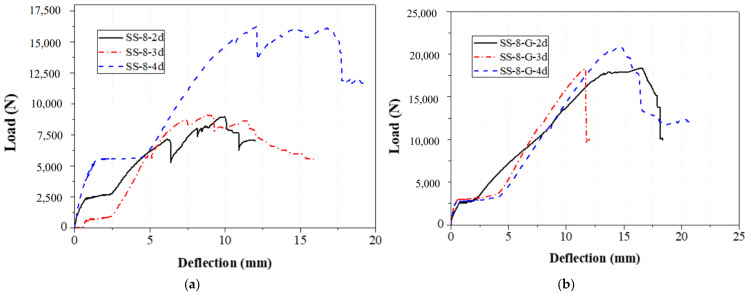
Load–displacement curve of 8 mm diameter specimen with double bolts; (**a**) without the gasket; (**b**) with the gasket.

**Figure 17:**
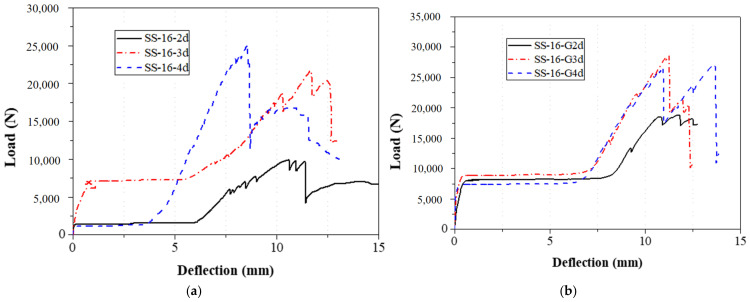
Load–displacement curve of 16 mm diameter specimen with double bolts; (**a**) without the gasket; (**b**) with the gasket.

The bolt diameters in the double-bolt tensile and shear tests were 8 mm and 16 mm, respectively. For each group, the edge distance varied from 1 d to 4 d. The load–displacement curves for 8 mm and 16 mm specimens are shown in Figure 16 and Figure 17, and failure modes are illustrated in Figure 10, Figure 11, Figure 12 and Figure 13. In the initial stage, all specimens exhibited a flat zone in the curve due to a gap between the bolt and clamp, which was difficult to eliminate. However, for sample SS-16-3d, the plateau appears at a higher load (~7 kN) compared to SS-16-2d and SS-16-4d (~2 kN). This difference may be attributed to reduced initial clearance or tighter bolt–hole engagement in the SS-16-3d specimen, which delays slip and causes earlier direct load transmission. Additionally, interaction effects between bolt spacing and edge distance may affect initial confinement and load path, resulting in a higher starting plateau. Following this, the load rose sharply until reaching the peak, after which it dropped rapidly due to local extrusion failure around the bolt holes. With continued loading, the curves showed a brief decline followed by a secondary rise and drop, ending with extrusion and shear failure at the end-side hole. Reinforced gaskets significantly improved the bearing capacity. When bolt diameter and edge distance remained constant, reducing the spacing between the two bolts enhanced the gasket’s effect. For 8 mm bolts, reducing the bolt spacing from 4 d to 2 d increased the load improvement from 36.79% (16.28 kN to 22.27 kN) to 129.15% (7.54 kN to 17.27 kN). For 16 mm bolts, the improvement was 81.59% (10.86 kN to 19.73 kN) at 2 d, but only 9.79% (25.64 kN to 28.15 kN) at 4 d.

Overall, the incorporation of reinforced gaskets greatly enhanced joint strength, especially under small end distances and bolt spacing. However, effectiveness diminished as spacing increased, emphasizing the importance of optimized geometric design when applying this strengthening strategy in practice.

#### 2.2.2. Effect of the Distance from Bolt Hole to the End

**Figure 18:**
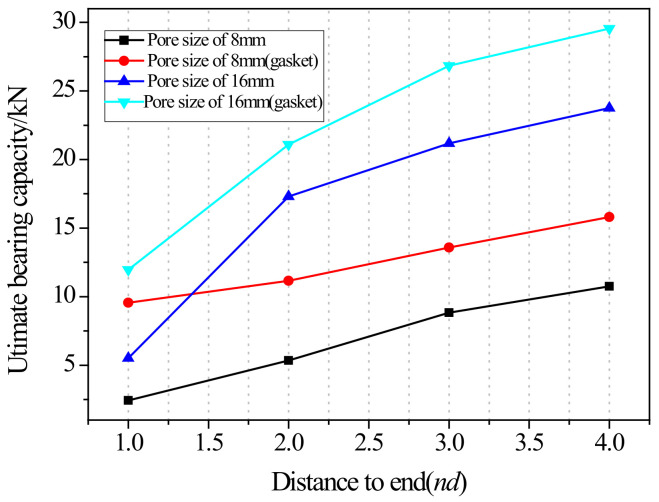
Ultimate bearing capacity regulation with the distance to the end of bolted joints on pultruded composite tubes.

The relationship of the ultimate bearing capacity regulation with the distance to the end of bolted joints on pultruded composite tubes is shown in Figure 18. When the bolt diameter is fixed, and the end distance changes from 1 d to 2 d, the ultimate load is sensitive to the change in end distance, and the ultimate load grows fastest relative to the end distance. This is particularly evident for the 16 mm bolt without a gasket, where the bearing capacity at 1 d is significantly lower due to severe stress concentration and premature failure near the bolt hole. When the end distance changed from 2 d to 4 d, the ultimate bearing capacity still increased, but the increasing magnitude decreased compared with the end distance from 1 d to 2 d, and the trend tended to be flat. The ultimate bearing capacity of two kinds of mechanical joints with different diameters had a similar trend with the changes in end distance. The effect of end distance on the ultimate bearing capacity was regular, which was an important factor affecting the ultimate bearing capacity. A qualitative analysis of the variation rule of ultimate bearing capacity was carried out, showing that the failure forms of joints transferred from shear failure to local extrusion failure. When the end distance was 1 d and 2 d, the failure forms were shear failures. The shear force that the joint can bear is basically proportional to the shear area of the joint plate. Local extrusion failure occurred when the end distance was 3 d. When the distance to the end was 4 d, almost all the failure forms were turned into local extrusion failure. These observations of failure transition from shear to local extrusion failure are consistent with the visual evidence shown in Figure 7, Figure 8, Figure 9, Figure 10, Figure 11, Figure 12 and Figure 13, where the evolution of damage modes under varying end distances is clearly depicted.

These results indicate a nonlinear relationship between end distance and joint strength, where initial increases yield significant improvements but further increases offer diminishing returns. The failure transition from shear to extrusion highlights the influence of load path evolution and stress redistribution.

#### 2.2.3. Effect of the Bolt Diameter

**Figure 19:**
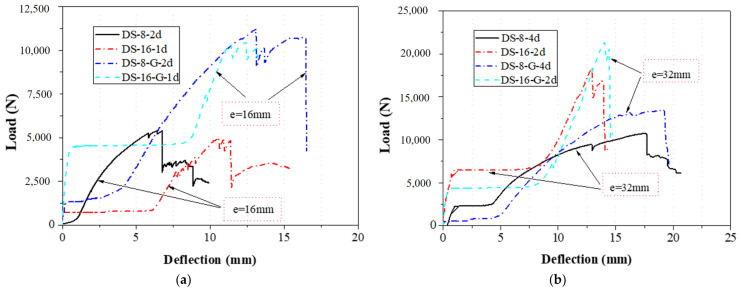
Load–displacement curve of bolt diameter; (**a**) end distance: 16 mm; (**b**) end distance: 32 mm.

As shown in Figure 19, the increase in the bearing capacity of the specimen was related to the size of the bolt diameter, and the bearing capacity increased with the increase in bolt diameter. When the end distance was 16 mm, the bearing capacity of the specimens without reinforced gaskets increased from 5.35 kN to 5.54 kN when the bolt diameter was 8 mm and 16 mm, respectively; the bearing capacity of the specimens with reinforced gaskets increased from 11.16 kN to 11.97 kN when the bolt diameter was 8 mm and 16 mm, respectively. When the end distance was 32 mm, the bearing capacity of the specimens without reinforced gaskets increased from 10.76 kN to 17.31 kN with bolt diameters of 8 mm and 16 mm, respectively; the bearing capacity of the specimens with reinforced gaskets increased from 15.81 kN to 21.10 kN, with bolt diameters of 8 mm and 16 mm, respectively. However, in configurations with shorter end distances (e.g., 16 mm), the increase in strength with larger bolt diameters was relatively modest. This may be due to local stress concentrations and inefficient stress redistribution near the edge, which constrain the full utilization of the bolt’s load-bearing potential.

While larger bolt diameters generally improved bearing capacity, the gains were more substantial at longer end distances. In short-end configurations, geometric constraints limited the full utilization of the bolt strength, suggesting that bolt diameter must be considered in conjunction with other design parameters.

## 3. Theoretical Analysis

### 3.1. Capacity of Single Bolt Joints

Shear failure is likely to occur when the distance from the bolt hole to the end is small, and the shear strength formula is defined by Equation (1).

(1) $$\tau =\frac{p}{2et}\leq [\tau_{\begin{array}{l} b \end{array}}]=\frac{\tau_{b}}{n}$$

where *t* is the thickness of the connection, *e* is the distance from the bolt hole to the end, *τ_b_* is the allowable shear strength of the joint, *n* is the security coefficient, and *p* is the load.

When the ratio of the section width to the diameter of the hole and the ratio of e to the diameter of the hole is large, the bolt is likely to generate stress concentration in the hole wall under the action of a large external load, resulting in extrusion failure. The extrusion strength formula is defined by Equation (2).

(2) $$\sigma_{j}=\frac{p}{dt}\leq [\sigma_{jb}],[\sigma_{jb}]=\frac{\sigma_{jb}}{n}$$

where $\sigma_{jb}$ is the extrusion strength of the material, $\left[\sigma_{jb}\right]$ is the allowable compression stress of the material, *n* is the security coefficient, and *p* is the load.

The joint is prone to splitting and damage because its transverse tensile strength is low. The split crack developed from the screw hole to the end of the specimen along the direction of the force. The splitter strength formula is defined by Equation (3).

(3) $$\sigma_{p}\leq [\sigma_{pb}]$$

where $\left[\sigma_{pb}\right]$ is the allowable stress of the material.

### 3.2. Capacity of Double Bolts Joints

In order to study the bearing capacity of porous bolt joints, Clarke and CNR [32,33] proposed a simple analysis model based on the design method of general composite bolt joints, in which the load distribution coefficient ηi accounts for the non-uniform force sharing among bolts. Under the corresponding failure mode, the joint load *P_u_* is defined by Equation (4).

(4) $$P_{u}=\frac{N_{i}P_{\mathrm{fc}}}{\eta_{i}}$$

where *N_i_* is the total number of bolt holes on row i, and i = 1, 2. *P*_fc_ is determined by the failure mode, and *η_i_* is the load distribution coefficient.

According to relevant research results [34,35,36], the load distribution is not consistent with the number of rows for multi-row bolts, while the load distribution coefficient of the same row bolts is a constant value. It can be verified by strain analysis that the value will not change with the dislocation of the bolt arrangement. Clarke [32] proved in the static tension test that 57% of the total tension was transferred to the first row of bolts and 43% to the second row of bolts. Shear action plays a leading role in influencing factors of ultimate bearing capacity, and the shear strength of a single bolt is defined by Equation (5).

(5) $$P_{\mathrm{fc}}=2\cdot f_{s}\cdot t\cdot e$$

where *f_s_* is the longitudinal shear length of the composite material, *e* is the characteristic shear length of the composite material, and *t* is the web thickness of the square tube.

### 3.3. Capacity of Joint with Gasket

In terms of the failure forms, the reinforcing gasket itself remained intact, but slight peeling at the specimen ends was visually observed. This local peeling, occurring away from the load transfer zone, had a negligible effect on the total bearing capacity. The total bearing capacity can be simplified as Equation (6).

(6) $$P_{z}=P_{b}+2v$$

where *P_z_* is the total bearing capacity of specimens, *P_b_* is the self-bearing capacity of specimens without gaskets, and *v* is the shear strength of structural adhesive at one end of the square tube.

### 3.4. Validation of Theoretical Prediction

Theoretical and experimental ultimate loads for specimens are compared in Table 6. For specimen DS-8-2d, the ultimate load of experimental and theoretical ultimate loads is 5.35 kN and 4.96 kN, respectively, and the deviation is −7.3%. The ultimate load of the experimental result is 1.97 kN for specimen SS-16-G-2d, which agrees well with the corresponding theoretical analysis result of 2.01 kN, and the deviation is 2.3%. The comparisons in Table 6 indicate that the theoretical ultimate loads agree with the experimental values. However, the theoretical and experimental values of a few specimens (DS-16-2d, DS-16-G-4d) differ greatly for the complexity and diversity of influencing factors. Due to the majority of the gaskets having a certain stripping, delamination, and bulging, most theoretical values are lower than experimental values for the specimens without gaskets, while most theoretical values are higher than experimental values for the specimens with gaskets.

In summary, the theoretical model provides reasonable predictions of the ultimate load, with deviations typically within an acceptable range. The observed discrepancies, particularly in specimens with reinforced gaskets, may stem from unmodeled effects such as partial peeling or interfacial delamination, which alter the actual stress transfer mechanisms. These results underscore the necessity of incorporating such phenomena in future modeling for improved accuracy.

## 4. Numerical Simulation and Discussion

### 4.1. Modeling Details

Numerical analysis of the tensile–shear behaviors of the composite square tube was carried out using a nonlinear explicit finite element (FE) software ANSYS/LS-DYNA v19.0. The top and bottom rigid plates were simplified as rigid plates simulated by *MAT_RIGID. The elastic–plastic material model *MAT_PLASTIC_KINEMATIC was employed to model the bolt. This is a cost-effective model to simulate the isotropic and kinematic hardening plasticity by taking the strain rate effects into account [37]. The specific values of the parameters used in this study are as follows: elastic modulus *E* = 2.1 × 10^5^ N/mm^2^, *E^tan^* = 1.18 × 10^3^ N/mm^2^, initial yield stress *σ*_0_ = 3.35 × 10^2^ N/mm^2^, Poisson’s ratio *μ* = 0.27, hardening parameter *β* = 0, and failure strain ε_f_ = 0.35. The Cowper–Symonds parameters adopted here are *C* = 40 and *p =* 5 [38]. A composite damage model (material type 22) was used to model the composite square tube. The orthotropic material with optional brittle failure for composites can be defined following the suggestion of Chang [39,40]. Table 7 lists the parameters for the composite square tube and gasket based on the composite damage model. The geometric nonlinearity was also considered in the simulation to accurately capture large deformation effects. The composite square tube and the gaskets were modeled as orthotropic materials using layered solid elements with through-thickness stacking. The laminate configuration was assigned in accordance with the actual lay-up used in the experimental specimens to ensure consistency between numerical simulation and physical testing. The layer definitions were implemented in the composite material model (*MAT_COMPOSITE_DAMAGE), allowing for an accurate representation of anisotropic mechanical behavior and progressive failure across the thickness direction.

### 4.2. Loading and Boundary Conditions

The quasi-static load was simulated by moving the top rigid plate with a constant upward velocity. The bottom rigid plate was constrained in all degrees of freedom, and the top rigid plate was fixed in all translational and rotational degrees of freedom except for the vertical displacement, which had a constant rate of 2 mm/min.

### 4.3. Contact Definition

The interactions between different components were defined using AUTOMATIC SURFACE_TO_SURFACE contact with a friction coefficient of 0.1 between the bolt and the inner wall of the hole. The bonding between the composite tube and the gasket was modeled using tied contact to simulate the structural adhesive layer. The interface between the tube and the rigid plates was defined using surface contact with hard contact in the normal direction and frictional behavior in the tangential direction. All connections were designed to reflect the experimental set-up as closely as possible.

The connection between the gasket and the composite square tube was simulated using *CONTACT_TIED_SURFACE_TO_SURFACE, assuming perfect bonding without interfacial failure. This approach enables accurate transmission of interfacial shear and normal forces without explicitly modeling adhesive degradation.

### 4.4. Validation and Discussion

**Figure 20:**
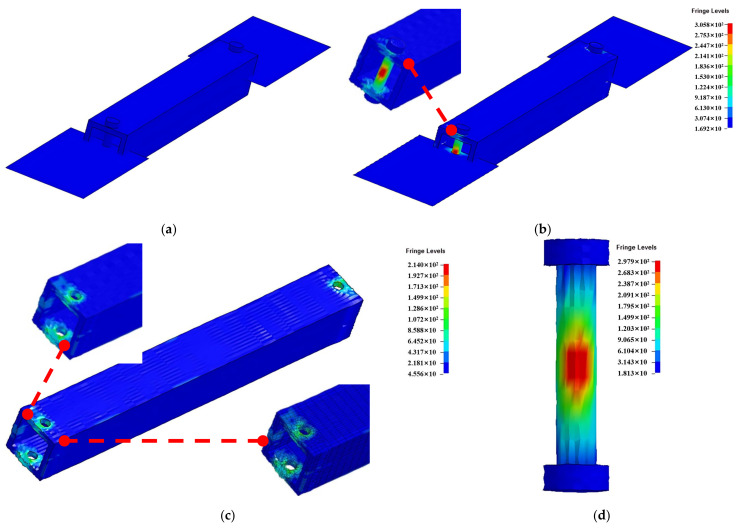
Numerical model of the tensile–shear test; (**a**) initial moment; (**b**) final moment; (**c**) simulation of the composite square tube; (**d**) simulation of the specimen with gasket.

**Figure 21:**
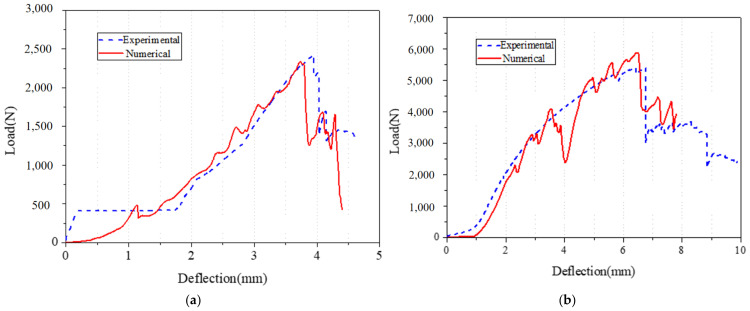
Comparison of experimental and numerical load–displacement curves; (**a**) specimen DS-8-1d; (**b**) specimen DS-8-2d.

Specimen DS-8-1d and DS-8-2d were selected for simulation. The failure mode is shown in Figure 20, and the fringe levels show the Von Mises stress of the composite square tube. The force–crush depth curve is shown in Figure 21. For specimen DS-8-1d, the peak force of the finite element simulation result is 2.31 kN, and the corresponding displacement is 3.79 mm. The results obtained from the numerical simulation agree well with the experimental result of 2.42 kN, with a deviation of the peak force of 4.6%. It was noted that the peak force obtained from the test occurs later than the numerical simulation. For specimen DS-8-2d, the peak force of the finite element simulation result is 5.85 kN, and the corresponding displacement is 7.83 mm. The experimental peak force is 5.35 kN, and the deviation of the peak force is 9.3%. It was noted that the peak force obtained from the test occurs later than the numerical simulation. As shown in Figure 21, load–displacement curves of the test and the finite element simulation both include two stages, the rising stage, and the plastic range. The statistical correlation (R^2^) between the experimental and numerical curves was calculated as 0.498 and 0.773 for DS-8-1d and DS-8-2d, respectively. This indicates that the numerical model provides a reasonably accurate prediction of the structural response trend, although some discrepancies remain due to local damage evolution and experimental variability.

## 5. Conclusions

This study investigated the bearing capacity and strengthening strategy of bolted joints in pultruded composite square tubes through experimental testing, theoretical analysis, and finite element simulation. The use of multiaxial fiber-reinforced composite gaskets was proposed to enhance joint performance. The main conclusions are as follows:

- Geometric effects (normalized): Geometric parameters had a significant impact on joint capacity. For single-bolt specimens without gaskets, increasing the normalized edge distance (e.g., from 1 d to 4 d) led to an increase in bearing capacity by a factor of 4.4 for 8 mm bolts and 4.3 for 16 mm bolts. For double-bolt specimens, increasing bolt spacing from 2 d to 3 d resulted in a capacity increase of over 100%, indicating strong sensitivity to spatial configuration;
- The introduction of multiaxial fiber-reinforced gaskets yielded up to a 295% improvement in load-bearing capacity in short edge distance scenarios. However, the effectiveness diminished as edge distance or bolt spacing increased. For instance, with 8 mm bolts, the enhancement dropped from 295% at 1 d to 47% at 4 d, revealing a clear trend of reduced marginal benefit with increasing clearance;
- As the edge distance increased, the dominant failure mode gradually shifted from shear failure to local extrusion failure. This transition highlights a nonlinear relationship between geometry and structural failure mechanisms;
- Theoretical predictions showed strong consistency with experimental results, with most peak load errors under 10%. Finite element simulations also demonstrated excellent agreement with test data, confirming the robustness of the numerical model;
- The use of multiaxial gaskets provides a practical and effective strengthening strategy for composite joints. Its advantages in stress redistribution and damage suppression make it well-suited for high-demand applications such as bridge nodes, aerospace fastener zones, and structural truss systems.

## Figures and Tables

**Table 1:** Details of composite pultruded square tubes with a single bolt.

Specimen	*L* (mm)	*t*_0_ (mm)	*t* (mm)	*d* (mm)	*e* (mm)
DS-8	300	5	0	8	8 (1 d); 16 (2 d); 24 (3 d); 32 (4 d)
DS-8-G	300	5	3	8	8 (1 d); 16 (2 d); 24 (3 d); 32 (4 d)
DS-16	300	5	0	16	16 (1 d); 32 (2 d); 48 (3 d); 64 (4 d)
DS-16-G	300	5	3	16	16 (1 d); 32 (2 d); 48 (3 d); 64 (4 d)

*t*_0_ is the thickness of the composite pultruded square tubes, *t* is the thickness of the gasket, *d* is the diameter of the bolt, and *e* is the distance from the bolt hole to the end.

**Table 2:** Details of composite pultruded square tubes with double bolts.

Specimen	*L* (mm)	*t*_0_ (mm)	*t* (mm)	*d* (mm)	*e* (mm)	*I* (mm)
SS-8	300	5	0	8	16	16 (2 d); 24 (3 d); 32 (4 d)
SS-8-G	300	5	3	8	16	16 (2 d); 24 (3 d); 32 (4 d)
SS-16	300	5	0	16	16	32 (2 d); 48 (3 d); 64 (4 d)
SS-16-G	300	5	3	16	16	32 (2 d); 48 (3 d); 64 (4 d)

*t*_0_ is the thickness of the composite pultruded square tubes, *t* is the thickness of the gasket, *d* is the diameter of the bolt, *e* is the distance from the bolt hole to the end, and *I* is the distance between the two bolt holes.

**Table 3:** Material properties.

Components	Properties	Sheets	Gaskets
Tension	Yield strength (MPa)	416.2	315.70
	Young’s modulus (GPa)	38.8	30.15
Compression	Yield strength (MPa)	337.27	223.74
	Young’s modulus (GPa)	20.92	16.01

**Table 4:** Failure mode and ultimate load of composite pultruded square tubes with a single bolt.

Specimen	*t* (mm)	*d* (mm)	*e* (mm)	Ultimate Load	Failure Mode
DS-8-1d	0	8	8 (1 d)	2.4	Shear failure
DS-8-2d	0	8	16 (2 d)	5.4	Shear failure
DS-8-3d	0	8	24 (3 d)	8.8	Shear failure
DS-8-4d	0	8	32 (4 d)	10.8	Shear and extrusion failure
DS-8-G-1d	3	8	8 (1 d)	9.6	Shear failure
DS-8-G-2d	3	8	16 (2 d)	11.2	Shear failure
DS-8-G-3d	3	8	24 (3 d)	13.6	Shear failure
DS-8-G-4d	3	8	32 (4 d)	15.8	Extrusion damage
DS-16-1d	0	16	16 (1 d)	5.5	Shear failure
DS-16-2d	0	16	32 (2 d)	17.3	Shear failure
DS-16-3d	0	16	48 (3 d)	21.2	Shear and extrusion failure
DS-16-4d	0	16	64 (4 d)	23.8	Extrusion damage
DS-16-G-1d	3	16	16 (1 d)	12.0	Shear failure
DS-16-G-2d	3	16	32 (2 d)	21.1	Shear failure
DS-16-G-3d	3	16	48 (3 d)	26.8	Extrusion damage
DS-16-G-4d	3	16	64 (4 d)	29.6	Splitter and extrusion damage

**Table 5:** Failure mode and ultimate load of composite pultruded square tubes with double bolts.

Specimen	*t* (mm)	*d* (mm)	*e* (mm)	*I* (mm)	Ultimate Load	Failure Mode
SS-8-2d	0	8	16	16 (2 d)	7.5	Shear failure
SS-8-3d	0	8	16	24 (3 d)	8.2	Shear failure
SS-8-4d	0	8	16	32 (4 d)	16.3	Shear failure
SS-8-G-2d	3	8	16	16 (2 d)	17.3	Shear failure
SS-8-G-3d	3	8	16	24 (3 d)	17.5	Extrusion damage
SS-8-G-4d	3	8	16	32 (4 d)	22.3	Extrusion damage
SS-16-2d	0	16	16	32 (2 d)	10.9	Shear failure
SS-16-3d	0	16	16	48 (3 d)	22.6	Splitter failure
SS-16-4d	0	16	16	64 (4 d)	25.6	Shear failure
SS-16-G-2d	3	16	16	32 (2 d)	19.7	Shear failure
SS-16-G-3d	3	16	16	48 (3 d)	29.2	Extrusion damage
SS-16-G-4d	3	16	16	64 (4 d)	28.2	Extrusion damage

**Table 6:** Comparisons between theoretical and experimental results for specimens.

Specimen	Ultimate Load (kN)		Deviation %	Specimen	Ultimate Load (kN)		Deviation %
	Experimental	Theoretical			Experimental	Theoretical	
DS-8-1d	2.42	2.48	2.5	DS-8-G-1d	9.56	7.32	−23.2
DS-8-2d	5.35	4.96	−7.3	DS-8-G-2d	11.16	13.37	20.4
DS-8-3d	8.83	7.44	−15.7	DS-8-G-3d	13.59	16.29	20.1
DS-8-4d	10.76	10.56	−11.1	DS-8-G-4d	15.81	19.32	22.6
DS-16-1d	5.51	4.96	−9.8	DS-16-G-1d	11.97	13.37	17.2
DS-16-2d	17.31	10.56	−38.9	DS-16-G-2d	21.10	19.32	−9.8
DS-16-3d	21.18	14.90	−29.3	DS-16-G-3d	26.84	32.65	22.6
DS-16-4d	23.76	19.90	−16.2	DS-16-G-4d	29.56	40.24	36.1
SS-8-2d	7.54	6.78	−10.3	SS-8-G-2d	17.27	15.23	−12.9
SS-8-3d	8.18	8.39	2.6	SS-8-G-3d	17.53	18.01	2.7
SS-8-4d	16.28	18.11	18.9	SS-8-G-4d	22.27	26.97	21.2
SS-16-2d	10.86	8.54	−21.6	SS-16-G-2d	19.73	20.12	2.0
SS-16-3d	22.56	22.89	1.5	SS-16-G-3d	29.19	28.47	−2.5
SS-16-4d	25.64	30.56	19.2	SS-16-G-4d	28.15	35.34	26.5

**Table 7:** Material parameters of cube and gasket.

Property	LS-DYNA Parameter	Experimental Value	
		Square Tube	Reinforcing Gasket
Density	R_O_	1.8 g/cm^3^	1.8 g/cm^3^
Modulus in the longitudinal direction	E_A_	38.8 GPa	30.15 GPa
Modulus in the transverse direction	E_B_	38.8 GPa	30.15 GPa
Modulus in the C direction	E_C_	8.12 GPa	7.68 GPa
Shear modulus	G_AB_	2.5 GPa	2.5 GPa
Shear modulus	G_AC_	1.5 GPa	1.5 GPa
Shear modulus	G_BC_	1.5 GPa	1.5 GPa
Poisson’s ratio	PRBA	0.15	0.15
Longitudinal tensile strength	XT	416.2 MPa	315.7 MPa
Transverse tensile strength	YT	416.2 MPa	315.7 MPa
Compressive strength in the longitudinal direction	XC	337.3 MPa	223.7 MPa
Compressive strength in the transverse direction	YC	337.3 MPa	223.7 MPa
Shear strength	SC	55 MPa	55 MPa

## Data Availability

The original contributions presented in this study are included in the article. Further inquiries can be directed to the corresponding author.
